# Coronary microvascular dysfunction and heart failure with preserved ejection fraction: what are the mechanistic links?

**DOI:** 10.1097/HCO.0000000000001082

**Published:** 2023-08-28

**Authors:** Aish Sinha, Haseeb Rahman, Divaka Perera

**Affiliations:** British Heart Foundation Centre of Excellence and National Institute for Health Research, Biomedical Research Centre at the School of Cardiovascular Medicine and Sciences, King's College London, London, UK

**Keywords:** coronary microvascular dysfunction, heart failure with preserved ejection fraction, lusitropy, nitric oxide, subendocardial ischaemia

## Abstract

**Purpose of review:**

Heart failure with preserved ejection fraction (HFpEF) accounts for half of all heart failure presentations and is associated with a dismal prognosis. HFpEF is an umbrella term that constitutes several distinct pathophysiological entities. Coronary microvascular dysfunction (CMD), defined as the inability of the coronary vasculature to augment blood flow adequately in the absence of epicardial coronary artery disease, is highly prevalent amongst the HFpEF population and likely represents one distinct HFpEF endotype, the CMD-HFpEF endotype. This review appraises recent studies that have demonstrated an association between CMD and HFpEF with an aim to understand the pathophysiological links between the two. This is of significant clinical relevance as better understanding of the pathophysiology underlying CMD-HFpEF may result in more targeted and efficacious therapeutic options in this patient cohort.

**Recent findings:**

There is a high prevalence of CMD, diagnosed invasively or noninvasively, in patients with HFpEF. Patients with HFpEF who have an impaired myocardial perfusion reserve (MPR) have a worse outcome than those with a normal MPR. Both MPR and coronary flow reserve (CFR) are associated with measures of left ventricular diastolic function and left ventricular filling pressures during exercise. Impaired lusitropy and subendocardial ischaemia link CMD and HFpEF mechanistically.

**Summary:**

CMD-HFpEF is a prevalent endotype of HFpEF and one that is associated with adverse cardiovascular prognosis. Whether CMD leads to HFpEF, through subendocardial ischaemia, or whether it is secondary to the impaired lusitropy that is characteristic of HFpEF is not known. Further mechanistic work is needed to answer this pertinent question.

## INTRODUCTION

Up to 50% of patients with angina have nonobstructive coronary arteries (ANOCA) [1]. ANOCA is an umbrella term comprising several distinct pathophysiological entities, the most common one being coronary microvascular dysfunction (CMD). Coronary microvascular dysfunction is defined as an inability of the coronary vasculature to augment coronary blood flow (CBF), in response to a physiological stressor, in the absence of obstructive epicardial coronary artery disease (CAD). An impaired coronary flow reserve (CFR), which is the ratio of maximal achievable flow and resting flow in response to adenosine-mediated vasodilatation, is the hallmark of CMD [2]. Heart failure can be dichotomized into reduced ejection fraction (HFrEF) and preserved ejection fraction (HFpEF). HFpEF is a heterogenous syndrome comprising several distinct underlying causes. It has a dismal prognosis, which is partly because of our incomplete understanding of its pathophysiology that in turn has led to a paucity in the therapeutic options available for these patients. Recent evidence has suggested a strong link between CMD and HFpEF, with up to 75% of patients with HFpEF having underlying CMD [3]. However, whether this link is causal or simply associative is currently not well understood and is the focus of several ongoing studies. This review article focuses on the pathophysiological links between CMD and HFpEF. 

**Box 1:**
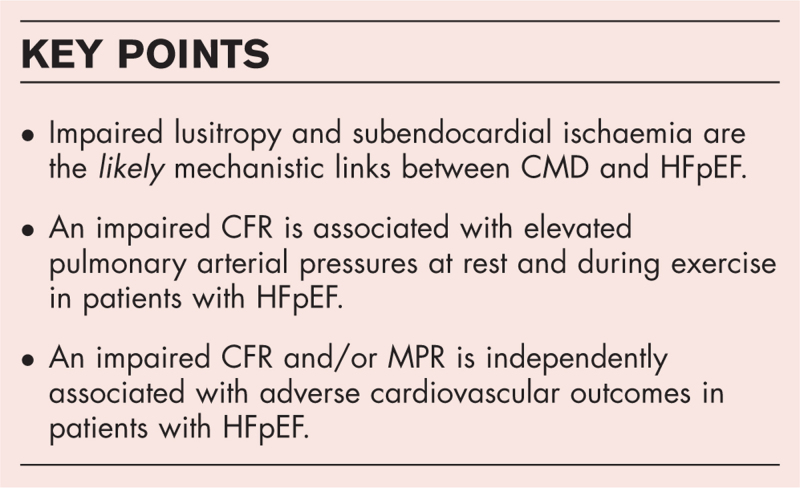
no caption available

## PATHOPHYSIOLOGY OF CORONARY MICROVASCULAR DYSFUNCTION

Coronary microvascular dysfunction can result from altered function of endothelial cells, leading to attenuated nitric oxide (NO) production (endothelium-dependent microvascular dysfunction), or it can be because of the inability of the vascular smooth muscle (VSM) cells to vasodilate in response to NO (endothelium-independent microvascular dysfunction) [4]. In clinical practice, the former is characterized by an inability to augment CBF by at least 50% in response to acetylcholine infusion, and the latter is characterized as CFR less than 2.5 [5] in response to adenosine-mediated vasodilatation. Indeed, both of these entities can, and frequently do, co-exist. Furthermore, a majority of patients who have endothelium-independent microvascular dysfunction also have endothelium-dependent microvascular dysfunction, suggesting that the latter may be a precursor to the former [4]. Common cardiovascular risk factors, such as hypertension, diabetes mellitus, hyperlipidaemia and cigarette smoking, are thought to lead to a systemic inflammatory state, which in turn leads to impaired endothelial and VSM cellular function. Up until recently, it was thought that endothelium-independent microvascular dysfunction was solely due to the inability to augment CBF during stress, that is, attenuation of hyperaemic flow. This was purported to be due to architectural changes, such as capillary rarefaction and vessel fibrosis. However, we have recently demonstrated that CMD may itself be a heterogenous condition comprising two distinct endotypes; functional and structural CMD [6]. Both endotypes are characterized by an impaired CFR and have similar degrees of coronary perfusion inefficiency during exercise and stress hypoperfusion on cardiac magnetic resonance (CMR) imaging; however, the underlying pathobiology appears to be distinct [6]. Patients with functional CMD have a normal minimal microvascular resistance and high resting CBF, whereas those with structural CMD have heightened minimal microvascular resistance and attenuated hyperaemic CBF [6]. Impairment of the NO pathway has been implicated in the pathophysiology of both these endotypes. The heightened resting CBF in functional CMD is purported to be due to elevated neuronal nitric oxide synthase (nNOS) levels, as nNOS regulates basal CBF [7], and the attenuated hyperaemic CBF in patients with structural CMD is purported to be due to impairment of the endothelial NOS (eNOS), as eNOS regulates CBF during stress [7]. These hypotheses are being investigated in ongoing studies. Therapeutic trials in patients with ANOCA have largely yielded equivocal results but whether endotype-directed therapy leads to better outcomes is currently not known.

## CORONARY MICROVASCULAR DYSFUNCTION AND HEART FAILURE WITH PRESERVED EJECTION FRACTION

### Demonstrable association

A close link has been observed between CMD and HFpEF over the past few years. The PROMIS-HFpEF study reported a 75% prevalence of CMD in patients with HFpEF, with an impaired coronary flow velocity reserve being associated with systemic endothelial dysfunction and elevated natriuretic peptide levels [3]. These findings have been corroborated by others since. Rush *et al.*[8] investigated hospitalized patients with HFpEF and reported the presence of CMD in 81% of those who did not have obstructive epicardial CAD. Arnold *et al.*[9^▪^] compared CMR-derived myocardial perfusion reserve (MPR) and markers of myocardial fibrosis between patients with HFpEF and controls. They reported an impaired MPR, defined as MPR less than 2, in 78% of patients with HFpEF compared with 48% of controls, as well as a correlation between MPR and left ventricular (LV) diastolic function and natriuretic peptide levels. They also demonstrated a link between MPR and 3-year outcomes in patients with HFpEF, whereupon those with a lower MPR had more HFpEF-related hospitalizations [9^▪^]. Similarly, Yang *et al.*[10] have found a strong association between CFR and noninvasive parameters of diastolic function (*E*/*A* ratio and *E*/*e′*). Again, patients with HFpEF who had an impaired CFR were at a higher risk of adverse cardiac outcomes compared with patients with HFpEF who had a normal CFR [10].

It is not known whether a specific CMD endotype predominates within the coronary microvascular dysfunction and heart failure with preserved ejection fraction (CMD-HFpEF) population. Mohammed *et al.*[11] have reported significant capillary rarefaction, which was associated with the degree of myocardial fibrosis, in an autopsy study of patients with HFpEF compared with controls. Interestingly, it has previously been demonstrated that capillary rarefaction may itself be caused by underlying coronary endothelium-dependent microvascular dysfunction [12], which is suggestive of a vicious cycle that may eventually lead to impaired diastolic function. Arnold *et al.*[9^▪^] also reported attenuated hyperaemic myocardial blood flow (MBF) as the driving force for impaired MPR in their HFpEF cohort, with no alterations in resting MBF. These studies suggest a link between structural CMD and HFpEF, with the hyperaemic blood flow being impaired because of either architectural changes within the coronary vasculature and/or impairment of the eNOS pathway. Srivaratharajah *et al.*[13] have also reported a high prevalence of impaired MPR in patients with HFpEF and nonobstructive coronary arteries. Their HFpEF cohort demonstrated both an elevated resting and attenuated hyperaemic MBF; the former was purported to be secondary to a heightened resting metabolic demand as supported by the significantly higher resting rate pressure product [13]. Other groups, such as Loffler *et al.*[14], have reported a heightened resting MBF as the key flow perturbation in patients with HFpEF. Importantly, in this study, there was no difference in LV mass between the groups, suggesting that the high resting MBF was not because of a higher myocardial oxygen demand secondary to greater LV mass [14]. Here, MPR was moderately correlated with pulmonary artery systolic pressure (PASP), that is, the lower the MPR the higher the PASP, and markers of myocardial fibrosis with lower MPR being associated with higher levels of myocardial fibrosis [14]. However, interestingly, no such link was observed between MPR and myocardial fibrosis in a later study by Arnold *et al.* although both MPR and markers of myocardial fibrosis were associated with adverse cardiovascular outcomes [9^▪^]. This raises the question as to whether the link between MPR and myocardial fibrosis is associative rather than causative [9^▪^]. Dryer *et al.* investigated 30 patients with HFpEF and compared them against 14 control patients. They split patients into four groups [normal CFR/normal index of microcirculatory resistance (IMR), normal CFR/high IMR, low CFR/normal IMR and low CFR/high IMR]; they reported a high prevalence of CMD (71%) in the HFpEF group, with higher right ventricular systolic and mean pulmonary pressures in those with an impaired CFR [15]. Whether HFpEF with low CFR/normal IMR and low CFR/high IMR are distinct pathophysiological entities or part of the same disease spectrum is not yet known. Finally, Ahmad *et al.*[16] investigated 51 patients with unexplained exertional dyspnoea (29 with HFpEF and 22 controls). They reported a high prevalence of coronary microvascular dysfunction in patients with HFpEF (46% had endothelium-independent microvascular dysfunction and 86% had endothelium-dependent microvascular dysfunction) [16]. Endothelium-independent microvascular function had an inverse correlation with rest and peak exercise left-sided cardiac filling pressures, whereas endothelium-dependent microvascular function had an inverse correlation with peak exercise left-sided cardiac filling pressures only [16]. The finding of elevated filling pressures during exercise in patients with CMD and HFpEF is suggestive of impaired lusitropy during exercise, which may be secondary to subendocardial ischaemia or represent a primary perturbation.

### Proposed mechanistic links

Two potential mechanisms, linking CMD and HFpEF, have been identified. These will be discussed in detail below.

#### Impaired lusitropy

**FIGURE 1:**
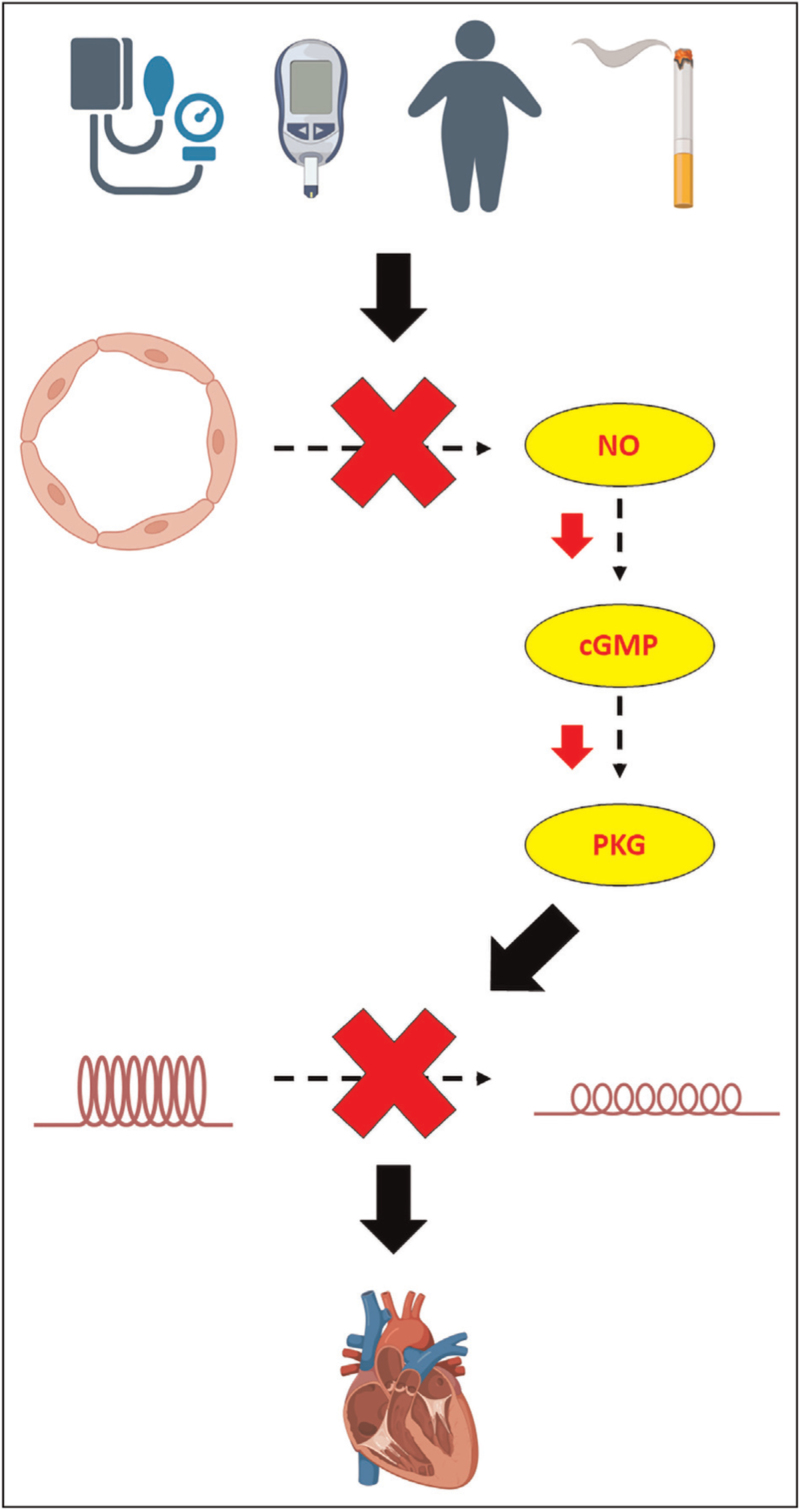
The role of the nitric oxide pathway in the pathogenesis of heart failure with preserved ejection fraction.

The novel paradigm of HFpEF pathophysiology implicates the NO pathway in HFpEF pathogenesis [17]. A systemic inflammatory state, induced by cardiovascular risk factors, leads to increased endothelial production of reactive oxygen species. Reactive oxygen species result in eNOS uncoupling and reduced NO production and bioavailability. This leads to a reduction in cyclic guanosine monophosphate (cGMP) and protein kinase G (PKG) activity. Protein kinase G is involved in titin phosphorylation; titin is a cytoskeletal protein that acts as a bidirectional spring and is responsible for early diastolic recoil and late diastolic distensibility of cardiomyocytes. Therefore, titin hypophosphorylation makes cardiomyocytes less compliant. The impaired NO–cGMP–PKG axis leads to heightened diastolic stiffness through hypophosphorylation of titin in cardiomyocytes, leading to impaired lusitropy and left ventricular diastolic reserve (Fig. 1).

Furthermore, an up-regulation of inducible nitric oxide synthase (iNOS) has also been implicated in the pathogenesis of HFpEF [18]. Pharmacological eNOS inhibition, in a murine model of HFpEF, led to enhanced iNOS activity. This led to dysregulation of protein quality control, resulting in accumulation of misfolded proteins, which ultimately resulted in cardiomyocyte dysfunction and impaired lusitropy. Importantly, pharmacological inhibition of iNOS improved lusitropy and exercise intolerance in this model of HFpEF [18]. Similarly, a recent murine model has alluded to the role of accentuated iNOS activity, secondary to cardiovascular risk factors such as obesity, in the development of CMD and LV diastolic dysfunction [19^▪^]. A high fat and high sucrose diet led to impaired MPR and LV diastolic dysfunction in this murine model, which were both reversed by iNOS antagonism. Furthermore, mice with deletion of iNOS-encoding gene did not develop an impaired MPR and diastolic dysfunction in response to a high fat and high sucrose diet [19^▪^].

These purported mechanistic links place the NO pathway at the centre of CMD-HFpEF pathogenesis.

#### Subendocardial ischaemia

Previous studies have demonstrated that patients with HFpEF have an inability to adequately augment their MBF during stress [20], which can lead to ischaemia [21]. Patients with HFpEF have reduced phosphocreatine : ATP ratio during exercise [22]. ATP is required during diastole for the detachment of the myosin head from actin. Reduced ATP production, because of inadequate myocardial perfusion, will likely lead to incomplete diastolic relaxation because of diastolic cross-bridge cycling. Repetitive cardiomyocyte injury, over time, may cause myocardial fibrosis leading to sustained rise in cardiac filling pressures not only during exercise but also at rest. A vicious cycle has been proposed, whereby attenuated oxygen delivery impairs myocyte relaxation, which promotes heightened myocardial tension and increased oxygen consumption demands [23].

### Interplay between lusitropy and subendocardial ischaemia (cardiac–coronary coupling)

**FIGURE 2:**
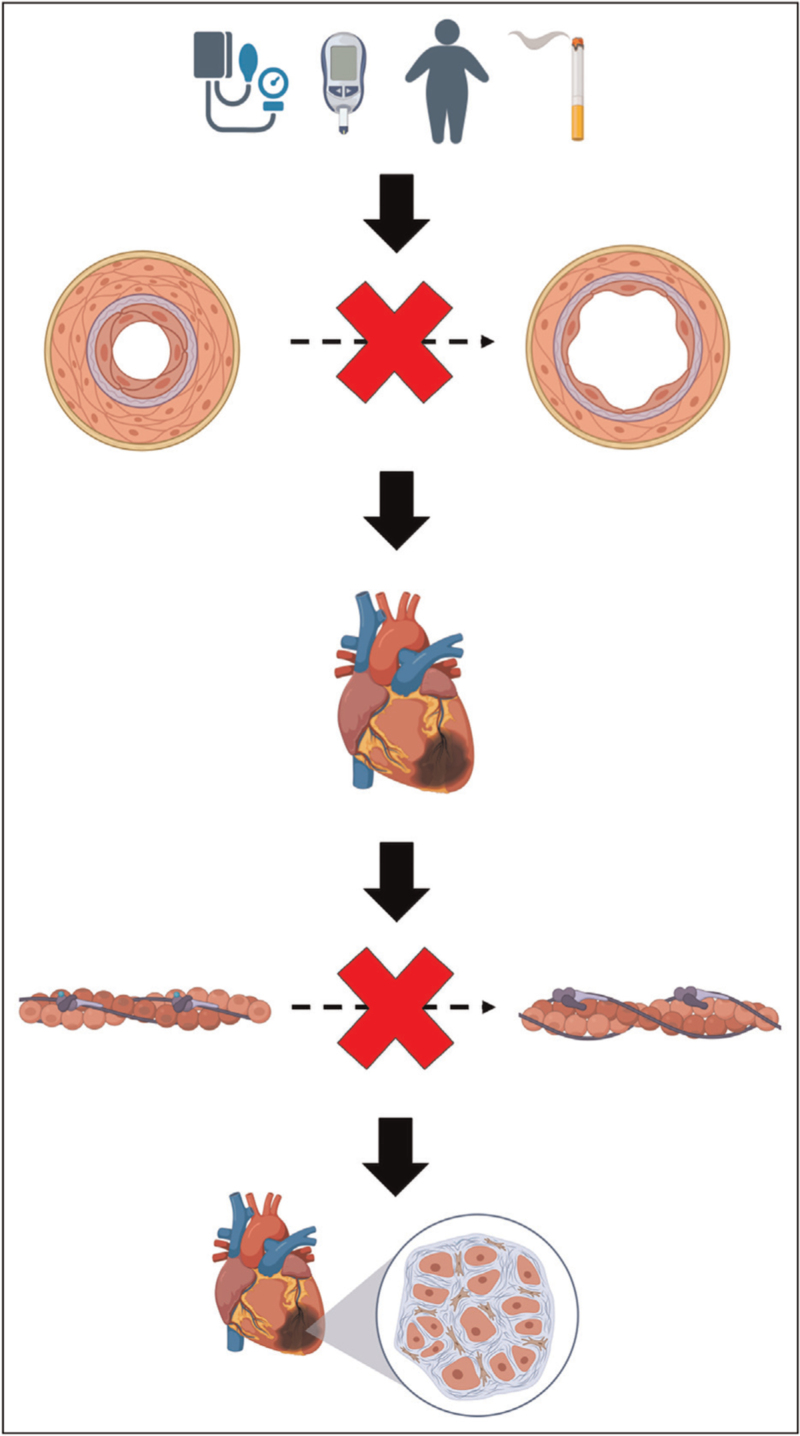
The role of coronary microvascular dysfunction and subendocardial ischaemia in heart failure with preserved ejection fraction.

Myocardial perfusion is dependent on CFR and the dynamic interaction between myocardium and microvasculature. Coronary flow is linked to myocardial relaxation and contraction; this process is called cardiac–coronary coupling, which can be characterized by coronary wave intensity analysis [24]. Wave intensity analysis defines the nature (accelerating or decelerating flow) and origin (aortic, designated as forward, or microcirculatory, designated as backward) of energy fluxes that govern CBF. The backward expansion wave (BEW) is the predominant wave in the healthy heart and is secondary to decompression of the microvasculature in early diastole. Therefore, it is directly related to the degree of ventricular relaxation (lusitropy) [25]. The major wave involved in flow deceleration is the backward compression wave (BCW), which arises during isovolumetric contraction. The relative balance of these wave energies defines the coronary perfusion efficiency, which is the proportion of accelerating energy in relation to total energy flux. Coronary perfusion efficiency and lusitropy are both enhanced during exercise in health [2,26]. In contrast, coronary perfusion efficiency decreases with exercise in CMD [2], primarily driven by attenuation of the accelerating BEW and accentuation of the decelerating BCW. In patients with CMD-HFpEF, the impaired lusitropy during exercise may be the primary driver of attenuated BEW, leading to subendocardial ischaemia. Alternatively, subendocardial ischaemia during exercise, secondary to CMD, may precipitate ischaemia-induced diastolic dysfunction, with diminished lusitropy subsequently impairing coronary perfusion further and resulting in a vicious ischaemic cascade. As coronary flow is intimately linked with myocardial relaxation, it can be challenging to ascertain causality between ischaemia and diastolic dysfunction, and perturbation of this relationship lies at the forefront of the CMD-HFpEF pathophysiology (Fig. 2).

## CONCLUSION

Coronary microvascular dysfunction is highly prevalent in patients with heart failure with preserved ejection fraction. CFR and MPR correlate with left ventricular diastolic function, LV filling pressures during exercise, and cardiac outcomes in patients with HFpEF. Impaired lusitropy and subendocardial ischaemia are the likely mechanistic links between CMD and HFpEF, although they are interlinked and identifying which comes first is often difficult to do. Further mechanistic work is needed to better characterize the CMD-HFpEF endotype with the end goal of better therapeutic options in this patient cohort.

## Acknowledgements

*None.*

### Financial support and sponsorship

*A.S. is the recipient of a UK Medical Research Council Clinical Research Training Fellowship grant (MR/T029390/1).*

### Conflicts of interest

*There are no conflicts of interest.*

## REFERENCES AND RECOMMENDED READING

Papers of particular interest, published within the annual period of review, have been highlighted as:

▪ of special interest
▪▪ of outstanding interest
